# High-Degree Neurons Feed Cortical Computations

**DOI:** 10.1371/journal.pcbi.1004858

**Published:** 2016-05-09

**Authors:** Nicholas M. Timme, Shinya Ito, Maxym Myroshnychenko, Sunny Nigam, Masanori Shimono, Fang-Chin Yeh, Pawel Hottowy, Alan M. Litke, John M. Beggs

**Affiliations:** 1 Department of Physics, Indiana University, Bloomington, Indiana, United States of America; 2 Santa Cruz Institute for Particle Physics, University of California at Santa Cruz, Santa Cruz, California, United States of America; 3 Program in Neuroscience, Indiana University, Bloomington, Indiana, United States of America; 4 MGH/HST Athinoula A. Martinos Center, Harvard Medical School, Charlestown, Massachusetts, United States of America; 5 Department of Neuroscience and Behavioral Disorders, Duke-NUS Graduate Medical School, Singapore; 6 Physics and Applied Computer Science, AGH University of Science and Technology, Krakow, Poland; The University of Texas at Austin, UNITED STATES

## Abstract

Recent work has shown that functional connectivity among cortical neurons is highly varied, with a small percentage of neurons having many more connections than others. Also, recent theoretical developments now make it possible to quantify how neurons modify information from the connections they receive. Therefore, it is now possible to investigate how information modification, or computation, depends on the number of connections a neuron receives (in-degree) or sends out (out-degree). To do this, we recorded the simultaneous spiking activity of hundreds of neurons in cortico-hippocampal slice cultures using a high-density 512-electrode array. This preparation and recording method combination produced large numbers of neurons recorded at temporal and spatial resolutions that are not currently available in any *in vivo* recording system. We utilized transfer entropy (a well-established method for detecting linear and nonlinear interactions in time series) and the partial information decomposition (a powerful, recently developed tool for dissecting multivariate information processing into distinct parts) to quantify computation between neurons where information flows converged. We found that computations did not occur equally in all neurons throughout the networks. Surprisingly, neurons that computed large amounts of information tended to receive connections from high out-degree neurons. However, the in-degree of a neuron was not related to the amount of information it computed. To gain insight into these findings, we developed a simple feedforward network model. We found that a degree-modified Hebbian wiring rule best reproduced the pattern of computation and degree correlation results seen in the real data. Interestingly, this rule also maximized signal propagation in the presence of network-wide correlations, suggesting a mechanism by which cortex could deal with common random background input. These are the first results to show that the extent to which a neuron modifies incoming information streams depends on its topological location in the surrounding functional network.

## Author Summary

We recorded the electrical activity of hundreds of neurons simultaneously in brain tissue from mice and we analyzed these signals using state-of-the-art tools from information theory. These tools allowed us to ascertain which neurons were transmitting information to other neurons and to characterize the computations performed by neurons using the inputs they received from two or more other neurons. We found that computations did not occur equally in all neurons throughout the networks. Surprisingly, neurons that computed large amounts of information tended to be recipients of information from neurons with a large number of outgoing connections. Interestingly, the number of incoming connections to a neuron was not related to the amount of information that neuron computed. To better understand these results, we built a network model to match the data. Unexpectedly, the model also maximized information transfer in the presence of network-wide correlations. This suggested a way that networks of cortical neurons could deal with common random background input. These results are the first to show that the amount of information computed by a neuron depends on where it is located in the surrounding network.

## Introduction

Networks of cortical neurons transmit and compute information. Fortunately, recent research has improved our understanding of both of these tasks [1–6]. Progress in these two areas lets us now ask how information modification (computation) depends on the number of connections a neuron receives (in-degree) or sends out (out-degree). This is an important question because almost nothing is currently known about how a neuron’s topological location in a complex network affects how it computes.

Connectivity has been widely studied in the brain at the macroscopic scale (see [1,2] for reviews) and at the microcircuitry or cellular level (e.g. [7–12]). At the cellular level, several studies have examined clustering in cortical networks and found that the networks are non-random [13–16]. In addition, several other studies have found or discussed the implications of a log-normal distribution of connection weights in networks of cortical neurons [17–22]. Finally, a few studies have provided evidence that the distribution of the number of connections made by a neuron (degree distribution) at the cellular level in hippocampus [23] and cortex [15,24] is heavy-tailed, which indicates the presence of neurons with many more connections than expected in a random network. These previous studies have provided valuable insights about information transmission between cortical neurons and they emphasize the need to better understand the structure of these networks.

Along these same lines, we recently used Transfer Entropy (TE) [25]–a well-established information theoretic method for detecting connectivity between neural sources [5,12,26–43]–to measure the time-scale dependent effective connectivity among hundreds of neurons in cortico-hippocampal slice cultures [44]. This analysis indicated the presence of time scale dependency in hub neurons, physical separation between connected neurons, and network modularity. The activity of these neurons was recorded at high temporal resolution (20 kHz) using a high density 512-electrode array (60 μm electrode spacing). This preparation and recording method combination yielded high neuron numbers at high temporal resolution, beyond what is currently available in any *in vivo* recording system. The temporal recording resolution of 50 μs was small enough to resolve synaptic delays of 1–20 ms that are typically found in cortex [45,46]. The interelectrode spacing of 60 μm also improved the likelihood of detecting synaptically connected neurons, which most often share contacts within a 200 μm radius [13,14]. Using the same data, in this work we sought to go *beyond* pairwise connectivity to examine the relationship between connectivity and computation among groups of individual neurons using newly developed tools from information theory.

It is a widely held position that neurons perform computations [5,47–54]. However, the precise definition of “computation” tends to vary throughout neuroscience and other fields that study systems that compute. Intuitively, computation involves combining information from different sources and producing some type of output. In contrast, communication or information transmission (the basis of many information theoretic neural connectivity studies) intuitively involves only passing information from a source to a receiver (see Materials and Methods – Information Theory Terminology). As a first step, in this novel analysis, we chose to use a recently introduced multivariate information theoretic tool (the Partial Information Decomposition (PID) [3,4]) to quantify the amount of information computed by a neuron about the firing states of other neurons that send it connections. We are aware of no other studies that have used this new tool to analyze computation among individual neurons. We chose to use the PID to measure computation because it allowed us to quantify in an information theoretic sense the amount of information processed by a neuron about the states of input neurons in distinct parts. One of these parts (the “synergy”) quantifies the bonus information processed by the receiver based on the non-overlapping information from both inputs simultaneously. Similar to [55], we feel it is natural to interpret the PID synergy as a measure of computation. We wish to emphasize that other definitions of “computation” exist and that other researchers have produced valuable and important studies using alternative definitions and analysis methodologies. We wish to make no claims that the PID synergy is the best or only measure of computation. Rather, we feel it is particularly well suited to analyzing computations performed by groups of neurons. Because the PID is an information theoretic analysis tool, it is able to capture linear and nonlinear interactions. Furthermore, unlike mutual information (which is a measure of information transmission or communication) or entropy (which measures the amount of information contained in an individual variable) [56], the PID is able to quantify the amount of information a neuron computed based on simultaneous inputs from other neurons.

In this analysis, we sought to determine if a neuron’s topological location in a complex network affected how it computes. To do so, we investigated if there were correlations between neuron degree and computation. Specifically, we addressed two main questions. First, did the in-degree of a neuron affect how much information it computed? For instance, did neurons that received many connections compute more information than neurons that received few connections? Second, did the out-degree of a neuron affect how much information was computed by the receivers of those connections? For instance, did neurons that received connections from high out-degree neurons tend to compute more information? In addition to these two main questions, we also examined the connection strength distribution and the degree distribution in our networks for comparison to previous results. Furthermore, we examined the role higher-order computations played in the network and we developed a simple feedforward network model to explore the relationship between computation and degree.

Using advanced tools from information theory and our high quality recordings of individual cortical neurons, we found the following results. (1) In agreement with previous studies, we found a roughly log-normal distribution of TE connection strengths and a heavy-tailed degree distribution. (2) The in-degree was independent of the amount of information computed by a neuron. Conversely, neurons that received connections from high out-degree neurons tended to compute more information than neurons that received connections from low out-degree. (3) Though higher-order computations are difficult to measure, we found evidence that higher-order computations did not dominate high in-degree neurons. (4) Using a simple feedforward network model as an illustrative example, we found that a degree-modified Hebbian model best matched computation/degree correlation results from the real data and simultaneously maximized signal propagation in the presence of network-wide correlations. Though the simplicity of our model implies it largely serves as a guide to future research, the model results do connect the issues of network topology and computation with the frequently discussed topic of signal propagation in correlated and noisy networks [57–63]. Previous versions of this work were presented at conferences in abstract form [64–66].

## Materials and Methods

### Ethics Statement

All neural tissue samples from animals were prepared according to guidelines from the National Institutes of Health and all animal procedures were approved by the Indiana University Animal Care and Use Committee (Protocol: 12–015) as well as the Animal Care and Use Committee at the University of California, Santa Cruz (Protocol: Litka1105).

### Experimental System

**Fig 1:**
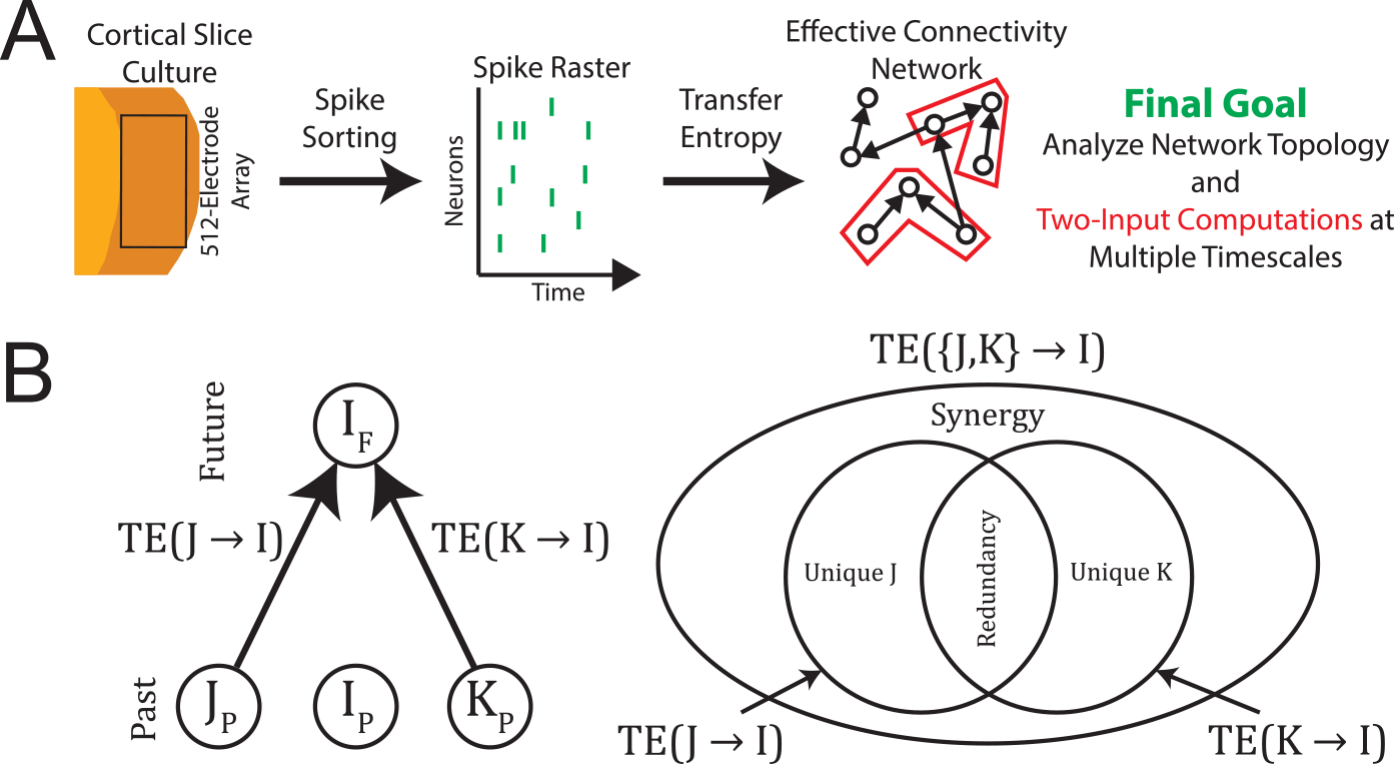
Analysis diagram and partial information decomposition. **(A)** We used a custom built 512-electrode array to record spiking activity from cortico-hippocampal organotypic cultures. We then used transfer entropy to detect effective connectivity among the recorded neurons. Finally, we studied the topology and the two-input computations in these networks. We also examined bounds on higher-order computation. **(B)** To study two-input computations, we used multivariate transfer entropy to deconstruct traditional transfer entropy measures into synergy, redundancy, and unique information terms [4]. Specifically, two-input computations were measured using the synergistic information computed for the system of two neurons sending significant amounts of information (as measured by transfer entropy) to a third neuron (see Materials and Methods – Multivariate Transfer Entropy and Computation).

A general overview of the analysis is presented in Fig 1A. The raw spiking data utilized in this analysis are fully described elsewhere [24,44]. Briefly, cortico-hippocampal organotypic cultures were produced using postnatal day 6 Black 6 mouse pups (wild-type C57BL/6 from Charles River) following the protocol described in [67]. The mice were anesthetized in an ice bath prior to decapitation and brain removal. Each culture was recorded after 2 to 4 weeks. After culturing, spontaneous activity was recorded from each slice using a custom made 512-electrode array system [68]. The array contained 5 μm diameter flat electrodes arranged in a triangular lattice with an inter-electrode distance of 60 μm. In this arrangement, the total recording area of the array was approximately a 0.9 mm by 1.9 mm rectangle. Though recordings from both hippocampus and cortex were performed [24,44], only recordings from cortex were used in this analysis (number of recordings: 25, recording region: somatosensory cortex). Action potentials (spikes) were then detected and spike-sorted using a well-established method (PCA using spike waveforms from seven adjacent electrodes) [24,44,68]. Duplicate neurons and neurons with many refractory period violations were then excluded from further analysis (see [68] for additional details). After spike sorting, neurons with less than 100 spikes in the 60 minute recording (firing rate < 0.028 Hz) were removed from the analysis. Spike sorting yielded spike times for each neuron (7735 total neurons) with a resolution of 50 μs. The average firing rate for each neuron was 2.10 Hz and each recording had an average of 309.4 neurons.

### Information Theory Terminology

One central purpose of information theory is to quantify information in spite of the natural ambiguity we associate with the concept of information. Throughout this paper, we have attempted to clearly articulate–both conceptually and mathematically–the information theoretic quantities we employed. Briefly, we wish to explicitly state and relate several important terms as we will use them. We will provide a full mathematical description of the measures in subsequent sections.

- Information: a general term that can apply to any type of information theoretic measure. In this analysis, all information theoretic quantities produced information results in units of bits, though the meaning or interpretation of those results were dependent upon the measure that was used to generate the results.
- Information transmission (or communication): the process of moving information between variables in time. In our analysis, it was typically measured using transfer entropy [25] or mutual information [56]. Mutual information and information transmission can also be considered forms of communication [56].
- Computation: a general term that has been widely applied [5,47–54] to situations in which information from two or more sources is combined to form some type of output. In this analysis, we identified a specific information theoretic measure (the partial information decomposition multivariate transfer entropy synergy [3,4]) as a measure of computation. We make no claim that this is the best or only way to mathematically define computation, rather that it is a reasonable interpretation of the information theoretic measure [55] that we utilized.

### Effective Connectivity Detection

Following the detection of action potentials, transfer entropy (TE) [25] was used to measure the effective connectivity between each pair of neurons. The complete procedure for the TE analysis is described in [44], though we provide a brief overview below. Several other methods of measuring information transfer or causality have been proposed in the past, including Granger Causality [69–77], Dynamic Causal Modeling [78–80], and directed information [81,82]. We chose to use TE because it has been widely used in neuroscience [5,26–43], it is model independent, it is capable of detecting nonlinear interactions, it is well suited to spiking data due to the discrete nature of spike trains and the need for discrete probability distributions in TE, and because it quantifies interactions in the general units of bits, which allows for straightforward comparisons between different systems (see [44] for more details). Herein, we used a subset of TE results produced in the previous analysis (see below).

In general, TE measures the amount of information the past state of one time series (call it *J*_*P*_) provides about the future state of another time series (call it *I*_*F*_) conditioned upon the past state of the receiver time series (call it *I*_*P*_). In our case, the time series were spike trains and the states were spiking or not spiking. Before defining the TE, it is first necessary to define mutual information (Eq 1) and conditional mutual information (Eq 2) [56]. Noting *p*(*i*_*F*_, *i*_*P*_, *j*_*P*_) as the probability for a given state (e.g. combination of spiking or not spiking) of the *I*_*F*_, *I*_*P*_, and *J*_*P*_ time series, the mutual information between *J*_*P*_ and *I*_*F*_, for instance, is given by:

$$MI\left(I_{F};J_{P}\right)=\sum_{i_{F},j_{P}}p\left(i_{F},j_{P}\right)\text{log}\left(\frac{p\left(i_{F},j_{P}\right)}{p\left(i_{F}\right)p\left(j_{P}\right)}\right)$$ (1)

The mutual information quantifies the amount of information one variable provides about the other. The mutual information is greater than or equal to zero and is symmetric (i.e. *MI*(*I*_*F*_; *J*_*P*_) = *MI*(*J*_*P*_; *I*_*F*_)). Mutual information can be used to quantify the communication between a source and a receiver [56]. As may be predicted by the name, the conditional mutual information is similar to the mutual information, except that it conditions the information shared between the original two variables by a third variable. The conditional mutual information between *J*_*P*_ and *I*_*F*_ conditioned on *I*_*P*_ is given by:

$$MI\left(I_{F};J_{P}|I_{P}\right)=\sum_{i_{F},i_{P},j_{P}}p\left(i_{F},i_{P},j_{P}\right)\text{log}\left(\frac{p\left(i_{F}|i_{P},j_{P}\right)}{p\left(i_{F}|i_{P}\right)}\right)$$ (2)

Then, the transfer entropy from J to I is simply defined as the conditional mutual information when the temporal relationship between variables described above is employed:

$$TE\left(J\to I\right)=MI\left(I_{F};J_{P}|I_{P}\right)=\sum_{i_{F},i_{P},j_{P}}p\left(i_{F},i_{P},j_{P}\right)\text{log}\left(\frac{p\left(i_{F}|i_{P},j_{P}\right)}{p\left(i_{F}|i_{P}\right)}\right)$$ (3)

In this analysis, the probability distributions were calculated by counting the number of occurrences of a given joint state throughout the hour long recording. Doing so required the assumption that the activity was stationary throughout the recording. Given the fact that spontaneous activity was recorded and that the cultures were isolated from outside stimuli, we feel this is an appropriate assumption. As described in [44], we normalized the TE by the entropy of the receiver:

$$TE{\left(J\to I\right)}_{Norm}=\frac{TE_{J\to I}}{-\sum_{i_{future}}p\left(i_{F}\right)\text{log}\left(p\left(i_{F}\right)\right)}$$ (4)

In our previous work, we used multiple bin sizes and delays to examine network connectivity at multiple discrete time scales, thus forming so called multiplex networks [44]. In this analysis, we used these same TE networks, but we chose to focus only on short time scales with interactions ranging from 1.6 to 6.4 ms and 3.5 to 14 ms because those time scales correspond well with the reported synaptic delay of 1–20 ms [45,46]. We chose to use overlapping time scales to ensure all interactions were captured. Note that a significant strength of this type of information theory analysis is that it is theoretically able to detect both excitatory and inhibitory interactions. However, because the neurons had relatively low firing rates, we predict that excitatory interactions were easier to detect because it is easier to detect a significant increase in an unlikely event than it is to detect a significant decrease in an unlikely event. We made no attempt to identify the excitatory or inhibitory interactions in the networks produced by our analysis.

To assess which connections were significant, we used a Monte Carlo approach to generate a null distribution of TE values [44]. This consisted of generating 5000 surrogate data sets for each pair of neurons using spike jittering and calculating their TE values. If less than 5 of the surrogate data sets produced TE values larger than the real data (i.e. p < 0.001), the connection was deemed significant. Finally, for each recording, 500 sub-networks with 50 neurons and average total degree 3 were produced from the full networks to reduce bias associated with average degree and network size [44,83].

### Multivariate Transfer Entropy and Computation

To move beyond bivariate connectivity and investigate information processing by neurons that receive inputs from two or more other neurons, it is necessary to employ a multivariate information measure. There has been a great deal of debate regarding recent developments in multivariate information theory [5,6], much of which is centered on the Partial Information Decomposition (PID) [3]. In addition, the PID has been used to establish a form of multivariate transfer entropy (Fig 1B, [4]). Though research is ongoing in this area and alternate methods have been put forward (see [5,6] for reviews, see [27,84–87] for examples), it is important to note that the PID multivariate TE possesses two distinct advantages over alternative methods, some of which have been used previously in neural system studies. First, PID multivariate TE can incorporate the necessary four variables (past states of three neurons plus the future state of one of the neurons) required to measure multivariate TE, unlike the other recently introduced multivariate information decompositions [84,86,87]. Second, PID multivariate TE can dissect the interaction into non-overlapping, non-negative terms, unlike the other multivariate TE methods [27,85] or previously used multivariate interaction methods, such as mutual information, entropy, or the interaction information [54,56]. Based on these advantages, we chose to employ the PID multivariate TE method.

Because the PID multivariate TE is rather complicated, we will only present a brief description here. The interested reader is directed to [3,4] for further details. Fundamentally, the PID can be thought of as a method for dissecting well defined information terms into relevant parts. For instance, if we have three time series (I, J, and K), we can consider the following decompositions (Fig 1B):

$$\begin{array}{c} TE\left(\left\{J,K\right\}\to I\right)=\text{Synergy}\left(\left\{J,K\right\}\to I\right)+\text{Unique}\left(K;J\to I\right)+\\ \text{Unique}\left(J;K\to I\right)+\text{Redundancy}\left(\left\{J,K\right\}\to I\right) \end{array}$$ (5)

TE(J→I)=Unique(K;J→I)+Redundancy({J,K}→I) (6)

TE(K→I)=Unique(J;K→I)+Redundancy({J,K}→I) (7)

In Eqs 5–7, we note {*J*, *K*} as a vector valued combination of time series J and K. In Eqs 5–7 we have used several information terms with intuitive meanings [3,4]. The unique terms correspond to the information provided only by that time series (J or K) about the future state of I. The redundant term corresponds to the information provided by both time series (J and K separately) about the future state of I. In Eq 6, for instance, note that the unique information from J depends on K. The TE from J to I is independent of K, but the redundant term in Eq 6 is dependent upon K. Therefore, the unique information from J is also dependent upon K. In other words, K influences what portion of the TE from J to I is redundant and what portion is unique. The synergistic term corresponds to the bonus information gained by the simultaneous knowledge of both time series (J and K together) about the future state of I. Note that all of the TE terms on the LHS of Eqs 5–7 can be calculated easily via Eq 3. If a method were found to calculate the redundant term, the unique terms could be calculated by subtracting the redundant term from the TE terms in Eqs 6 and 7. Then, the synergy term could be found by subtracting the redundant and unique terms from the TE term in Eq 5.

Thankfully, Williams and Beer provide a method for measuring the redundant term in Eqs 5–7 [3,4]. They define the redundancy using a quantity called the minimum information *I*_min_:

$$\begin{array}{l} \text{Redundancy}\left(\left\{J,K\right\}\to I\right)\equiv I_{\text{min}}\left(I_{F};J_{P},K_{P}|I_{P}\right)=\\ \;\sum_{i_{F}}p\left(i_{F}\right){\text{min}}_{R\in \left\{J_{P},K_{P}\right\}}I_{spec}\left(I_{F}=i_{F};R|I_{P}\right)=\\ \;\sum_{i_{F}}p\left(i_{F}\right){\text{min}}_{R\in \left\{J_{P},K_{P}\right\}}\left[I_{spec}\left(I_{F}=i_{F};R,I_{P}\right)-I_{spec}\left(I_{F}=i_{F};I_{P}\right)\right] \end{array}$$ (8)

where the specific information *I*_*spec*_ is given by:

$$I_{spec}\left(I_{F}=i_{F};R,I_{P}\right)=\sum_{r,i_{P}}p\left(r,i_{P}|i_{F}\right)\text{log}\left(\frac{p\left(r,i_{P},i_{F}\right)}{p\left(r,i_{P}\right)p\left(i_{F}\right)}\right)$$ (9)

and

$$I_{spec}\left(I_{F}=i_{F};I_{P}\right)=\sum_{i_{P}}p\left(i_{P}|i_{F}\right)\text{log}\left(\frac{p\left(i_{P},i_{F}\right)}{p\left(i_{P}\right)p\left(i_{F}\right)}\right)$$ (10)

The minimum information can be thought of as a weighted sum of the common information from time series J and K about each state of the future of time series I (i.e. the information provided by both J and K individually about each state of the future of time series I), conditioned upon the past state of time series I. Thus, it is the shared information provided by time series J and K, and therefore can be viewed as the redundancy. We calculated the redundancy using Eqs 9 and 10, as well as the transfer entropy using Eq 3. We then used the relationships given in Eqs 5–7 to calculate the unique information terms and the synergistic information. We normalized the information terms using the entropy of the future state of I, identical to the procedure described by Eqs 3 and 4 for normalizing TE.

**Fig 2:**
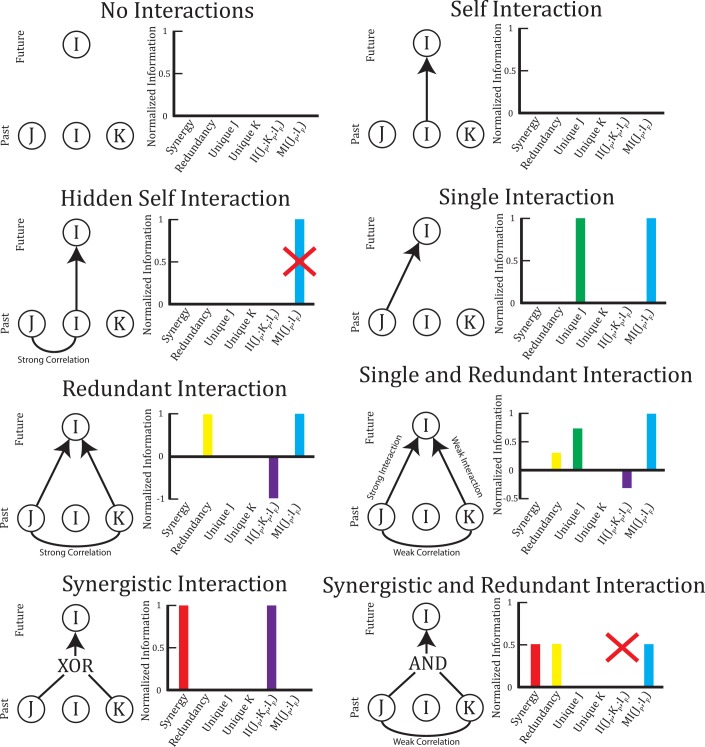
Example multivariate TE interactions. The PID multivariate TE is able to dissect different types of computations performed by one receiving neuron (I) with two transmitting neurons (J and K). Unique information is the portion of the information provided by one transmitter alone, redundancy is the portion of information provided by both transmitters, and synergy is the portion provided only by the combined input of both transmitters. Note that mutual information *MI*(*J*_*P*_; *I*_*F*_) does not detect common drive from the history of the receiving neuron (Hidden Self Interaction Example, red X). Note that the interaction information *II*(*J*_*P*_; *K*_*P*_; *I*_*F*_) is not able to detect simultaneous synergy and redundancy (Synergistic and Redundant Interaction Example, red X) and it is not able to detect unique information (Single and Redundant Interaction Example).

The terms produced by the PID multivariate TE analysis can be best understood using several example systems (Fig 2, Table 1). To aid in the comparison with previously used information theory measures, we also calculated the interaction information *II*(*J*_*P*_; *K*_*P*_; *I*_*F*_) (Eq 11) [6,54,88] between the transmitting time series and the receiver, as well as the mutual information between one of the transmitting time series and the receiver *MI*(*J*_*P*_; *I*_*F*_) (Eq 1) [56] in these simple examples.

$$II\left(J_{P};K_{P};I_{F}\right)=II\left(\left\{J,K\right\}\to I\right)=MI\left(J_{P};K_{P}|I_{F}\right)-MI\left(J_{P};K_{P}\right)$$ (11)

**Table 1:** Probability distributions for example multivariate TE interactions. This table contains the joint probability distributions used to create the example interactions shown in Fig 2.

Variables				p(I_f_,I_p_,J_p_,K_p_)							
I_F_	I_p_	J_p_	K_p_	No Interaction	Self Interaction	Hidden Self Interaction	Single Interaction	Redundant Interaction	Single and Redundant Interaction	Synergistic Interaction	Synergistic and Redundant Interaction
0	0	0	0	1/16	1/8	1/4	1/8	1/4	0.1964	1/8	0.0548
0	0	0	1	1/16	1/8	1/4	1/8	0	0.0536	0	0.0548
0	0	1	0	1/16	1/8	0	0	0	0	0	0.0548
0	0	1	1	1/16	1/8	0	0	0	0	1/8	0
0	1	0	0	1/16	0	0	1/8	1/4	0.1964	1/8	0.0548
0	1	0	1	1/16	0	0	1/8	0	0.0536	0	0.0548
0	1	1	0	1/16	0	0	0	0	0	0	0.0548
0	1	1	1	1/16	0	0	0	0	0	1/8	0
1	0	0	0	1/16	0	0	0	0	0	0	0
1	0	0	1	1/16	0	0	0	0	0	1/8	0
1	0	1	0	1/16	0	0	1/8	0	0.0536	1/8	0
1	0	1	1	1/16	0	0	1/8	1/4	0.1964	0	0.3357
1	1	0	0	1/16	1/8	0	0	0	0	0	0
1	1	0	1	1/16	1/8	0	0	0	0	1/8	0
1	1	1	0	1/16	1/8	1/4	1/8	0	0.0536	1/8	0
1	1	1	1	1/16	1/8	1/4	1/8	1/4	0.1964	0	0.3357

In these simple examples (Fig 2, Table 1), note that the mutual information does not detect common drive from the history of the receiving neuron (Hidden Self Interaction Example). The interaction information is not able to detect simultaneous synergy and redundancy (Synergistic and Redundant Interaction Example) and it is not able to detect unique information (Single and Redundant Interaction Example). The simultaneous synergy and redundancy example is especially relevant in neural networks if we assume that input neurons are usually somewhat correlated and the receiver neuron functions like an integrate-and-fire neuron (i.e. similar to an AND-gate). Thus, the ability of the PID multivariate TE to separate out synergistic and redundant portions is a crucial advantage over previous multivariate methods that are unable to make such a separation.

Following the dissection of multivariate TE into synergistic, redundant, and unique components using the PID, we felt it was appropriate to identify the synergistic term as a measure of computation performed by the receiver neuron. This is a natural interpretation of the synergistic term because synergy requires simultaneous knowledge of the states of both input neurons and non-trivial computation intuitively requires the combination of at least two pieces of information. This interpretation can be further explained using the examples in Fig 2. Synergy is only found in the Synergistic Interaction example and the Synergistic and Redundant Interaction example. These are the only examples where neuron I utilizes simultaneous knowledge of J and K to determine its state. Other examples, such as the Single Interaction example, show cases where I and J share information (e.g. I in the future predicts J in the past or vice versa), but no computation is present. In this sense, the synergy quantifies the degree to which I required simultaneous knowledge of J and K in these complex multivariate temporal relationships and we believe it is reasonable to interpret it as a measure of computation. A similar identification between synergy and information modification has been asserted previously [55]. To be clear, in this work we wish to make no claim that the PID multivariate TE synergy term is the best or only measure of computation. Rather, we feel defining computation using synergy is a natural and reasonable interpretation.

In addition, we feel it would not be appropriate to define computation in an information theoretic sense using the joint entropy between the neurons or the time lagged mutual information, for instance. The joint entropy simply calculates the overall variability of the variables, which is different from computation. Time lagged mutual information would provide a description of information flow, but it is not able to quantify the outcome of information being combined from two sources, as is done in the PID.

**Fig 3:**
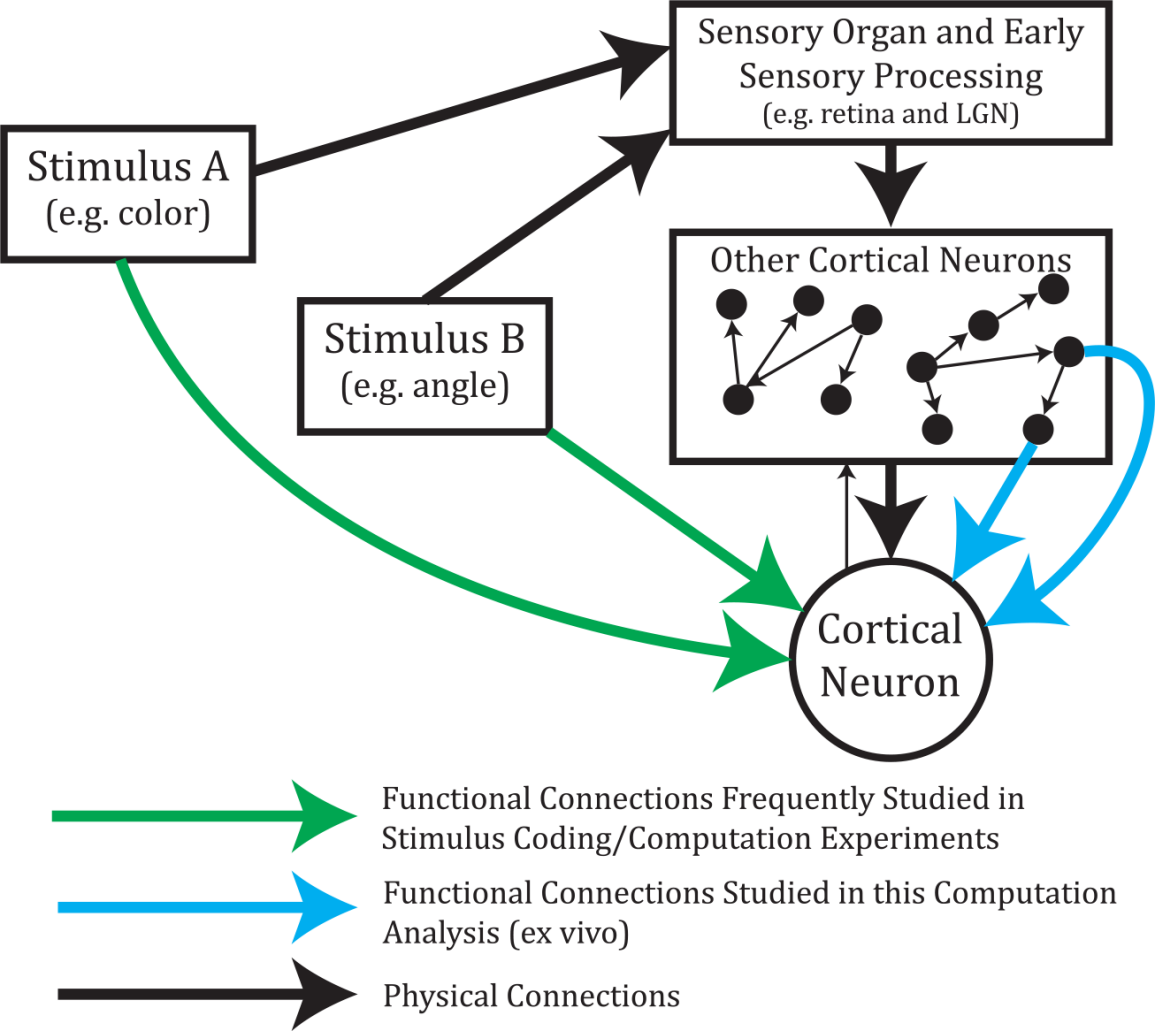
Computations were performed by neurons using information about the spiking state of other neurons. Many previous studies have examined the ability of neurons to compute information about stimuli via functional connections from those stimuli to cortical neurons (green arrows). In our analysis, we examined the computations performed by neurons about the spiking states of functionally connected neurons (blue arrows). Also, note that our experimental system is *ex vivo* and we analyzed spontaneous activity.

Furthermore, we wish to clarify that our definition of computation is based solely on the spiking activities of neurons. In other words, when we say one neuron is computing information from other neurons, the information being computed is represented by the input neurons’ spike trains, not some sensory stimuli or other non-neuronal signal. A great deal of previous research has focused on computation and coding in the brain related to sensory stimuli (e.g. [49]) and we wish to highlight the conceptual difference between our work and those previous works (see Fig 3). Note that we make no assumptions about the types of computations or operations the receiving neuron performs based on the input neurons’ spike states. Information theory is capable of detecting linear and nonlinear interactions, making it ideal for this type of study. The computation calculated as the PID multivariate TE synergy term for the two-input system (two input neurons and one receiver neuron) formed the majority of this analysis.

Following the detection of significant bivariate connections using TE (described above), the PID multivariate TE synergy term (computation) was calculated for all possible groups of three neurons such that two of the neurons sent significant TE connections to the third neuron. The binning and delay methods for the PID multivariate TE were identical to the bivariate TE analysis [44]. This implied that the previously calculated bivariate TE values were identical to the bivariate TE terms in Eqs 6 and 7.

Using TE and the PID multivariate TE to analyze systems of two or three neurons was possible using the methods described above because each systems was assumed to be isolated. However, calculating higher-order synergy (computation) terms for systems with more than two transmitting neurons becomes computationally difficult because the number of PID terms grows very rapidly as the number of variables increases [3]. However, it is relatively easy to calculate a bound on the highest order synergy term. The highest order synergy term must be less than or equal to the information gained by including an additional input:

$$\begin{array}{l} I_{gain}\left(I_{F};I_{P},J_{1,P},\ldots ,J_{n-1,P};J_{n,P}\right)=\\ \;TE\left(\left\{J_{1,P},\ldots ,J_{n,P}\right\}\to I_{F}\right)-TE\left(\left\{J_{1,P},\ldots ,J_{n-1,P}\right\}\to I_{F}\right)\geq \text{Synergy}\left(\left\{J_{1},\ldots ,J_{n}\right\}\to I\right) \end{array}$$ (12)

The information gain bounds the highest order synergy because the highest order synergy will only be present in the highest order TE term (left TE term in Eq 12) and not in the TE from all but one of the inputs (right TE term in Eq 12). Subtracting the lower order TE will remove many of the lower order terms, but leave the highest order synergy and other higher-order terms. For a group of *n* input neurons, we averaged the information gain across the *n* possible permutations of input neurons. We calculated the information gained for up to six input connections. For each receiver and value of n input neurons, we calculated the information gain for either all possible combinations of inputs or 100 sample combinations, whichever was smaller. We chose these parameters because they are large enough to adequately sample the data and convey general trends in the data, but small enough to allow for a reasonable computation time.

### Feedforward Network Model

We used a simple feedforward network to see if a given synaptic wiring rule could reproduce the patterns of connectivity and synergy we found in the *in vitro* data. The model contained very few neurons and was meant to represent a small segment of a larger network. The activity of the larger portion of the network was approximated using a simple binary signal. This model was constructed to provide clear connections to the computation results found in the biological networks and to motivate future research with more sophisticated models. The model’s simple structure and illustrative purpose imply that conclusions drawn from it cannot be directly applied to biological networks, but rather should serve as guides to develop new hypotheses.

The model had two layers (referred to as the input and output layers), each with 20 binary state neurons. We decided to use only 20 neurons to reduce computation time and because other neurons in the network were approximated with the binary signal (see below). The neurons functioned using a probabilistic rule that used a sigmoid function to define the likelihood *p* for the neuron to fire given the total amount of input current *I*:

$$p\left(I\right)=\frac{1}{1+e^{-\alpha I+\beta }}$$ (13)

We utilized a sigmoid function to define the firing probability to introduce nonlinear behavior in the neurons. Furthermore, the sigmoid function produced the desired general behavior that low currents should cause the neuron to spike infrequently, high currents should cause the neuron to spike frequently, and mid-range currents should produce approximately linear changes in firing probabilities. Sigmoid functions have been widely used in the neural network literature in a variety of applications to introduce similar non-linear neuron behavior (e.g. [89–91]). In Eq 13, the constants *α* and *β* were identical for all neurons and set via the following conditions:

p(I=0)=0.01 (14)

$$p\left(I=I_{con}\;*\;N_{Neurons}\right)=0.5$$ (15)

In Eq 15, *I*_*con*_ represented the amount of current carried by each connection from the input layer to the output layer (see below) and *N*_*Neurons*_ represented the number of neurons in the input and output layers (i.e. *N*_*Neurons*_ = 20). The probability in Eq 14 was chosen to ensure that neurons with no input current would rarely fire spontaneously. The probability in Eq 15 was chosen to produce separate firing regimes for maximally connected output layer neurons (see below). These conditions produced values of *α* ≈ 0.023 and *β* ≈ 4.6.

To approximate network-wide activity, all neurons received input current from a random binary signal (*b*(*t*) = 0,1) whose states were equally likely. Note that the binary signal was included not to model some type of external stimulus because our experimental system utilized only spontaneous activity. Rather, the binary signal was used to model large scale changes in other neurons that were not explicitly considered in the model. As an example of the type of network-wide changes that the binary signal could be considered to model, many of the cultures in our experiments showed bursts of elevated activity [44].

The relationship between the current from the binary signal was not uniform for all neurons. This produced varying degrees of correlation between neuron activity and the binary signal. The current was varied to produce a linear change in firing probability in the absence of connectivity across neurons based on the binary signal state. Specifically, we used the following relationship for the i^th^ input layer neuron to establish the appropriate current via Eq 13:

$$p\left(b\left(t\right)=0,i\right)=0.5-0.4\frac{i-1}{N_{Neurons}-1}$$ (16)

$$p\left(b\left(t\right)=1,i\right)=0.5+0.4\frac{i-1}{N_{Neurons}-1}$$ (17)

For the o^th^ output layer neuron, we used similar relations:

$$p\left(b\left(t\right)=0,o\right)=0.5-0.2\frac{o-1}{N_{Neurons}-1}$$ (18)

$$p\left(b\left(t\right)=1,o\right)=0.5+0.2\frac{o-1}{N_{Neurons}-1}$$ (19)

The parameter values in Eqs 16–19 were chosen for several reasons. In the absence of connectivity, the first neurons in the input and output layer were uncorrelated with the binary state and equally likely to be active or inactive, while the last neurons were strongly correlated with the binary state. The correlation was reduced for the output layer to allow for influence from connectivity.

Connectivity was established in the network by randomly inserting $N_{Connections}=0.2\;*\;N_{Neurons}^{2}=80$ binary connections from input layer neurons to output layer neurons. By fixing the connectivity density in the network at 0.2, we insured that, in the randomly connected version of the network, each input layer neuron connected to roughly one-fifth of the output layer neurons. Each run of the network was independent and consisted of the following steps: (1) Randomly select the binary signal value. (2) Inject the appropriate current into the input layer neurons based on the binary signal value. (3) Determine the spiking state of the input layer neurons based on the probabilistic rule. (4) Inject 10 units of current (*I*_*con*_ = 10) into output layer neurons based on active connections from input layer neurons. (5) Inject the appropriate current into the output layer neurons based on the binary signal value. (6) Determine the spiking state of the output layer neurons based on the probabilistic rule and the injected current from the binary signal and the connectivity.

For several models, the initial random connectivity was altered using a Hebbian or modified Hebbian rule. The rewiring proceeded by running the network 500 times to gather statistics and then calculating a rewiring score *S*(*i*, *o*) for each pair of neurons in the input and output layers. The connected pair with the lowest score was disconnected and the unconnected pair with the highest score was connected. This process of gathering statistics, calculating the rewiring score, and performing one rewiring was performed *N*_*Connections*_ times. For each unique type of model, 100 example models were produced.

In general, the score was calculated as:

$$S\left(i,o\right)=A\left(i,o\right)+a_{1}Deg_{In}\left(o\right)+a_{2}Deg_{Out}\left(i\right)+a_{3}FR\left(o\right)+a_{4}FR\left(i\right)$$ (20)

In Eq 20, *A*(*i*, *o*) was the proportion of network runs that produced identical states between the i^th^ input layer neuron and the o^th^ output layer neuron (i.e. the agreement between the neurons). *Deg*_*In*_(*o*) and *Deg*_*Out*_(*i*) were the in-degree of the o^th^ output layer neuron and the out-degree of the i^th^ input layer neuron, respectively. To the best of our knowledge, neuron degree has not previously been explicitly incorporated in Hebbian wiring rules, though the relationship between wiring and degree in many types of networks has been studied in terms of preferential attachment (e.g. [92]). We felt it would be interesting to include these factors given the fact that we investigated the relationship between computation and neuron degree. *FR*(*o*) and *FR*(*i*) were the firing rates of the o^th^ output layer neuron and i^th^ input layer neuron, respectively. *a*_1_, *a*_2_, *a*_3_, and *a*_4_ were parameters that could be set to produce different types of rewiring rules and thus different types of models.

In this analysis, we examined four types of models. The first model utilized the random initial connectivity and no rewiring was performed. The second model used *a*_1_ = *a*_2_ = *a*_3_ = *a*_4_ = 0 and, therefore, employed a purely Hebbian rewiring rule. The third and fourth models were restricted to *a*_3_ = *a*_4_ = 0 and *a*_1_ = *a*_2_ = 0 to produce degree-modified and firing rate modified Hebbian rewiring rules, respectively. The precise values of the non-zero parameters in these two models were set via a three step manual lattice search of parameter space (bounds: −3 ≤ *a*_1_, *a*_2_ ≤ 3, −4 ≤ *a*_3_, *a*_4_ ≤ 4) to find the model that most closely matched the computation vs. degree correlation results seen in the real data. Therefore, the rewiring rules were themselves fits to the data and the resulting parameter values represented important results (See *Results – Feedforward Network Model*section below for further details on the final model parameter values).

To insure that results from the model were not heavily dependent on the number of neurons, we ran the model with 40 neurons each in the input and output layers (double the original size, *N*_*Neurons*_ = 40). All other parameters and equations related to the model were used as defined above. A similar three step manual lattice search of score parameters space was used to find score parameters for the degree-modified and firing rate modified Hebbian rewiring rules.

## Results

### Connection Weight and Degree Distributions

**Fig 4:**
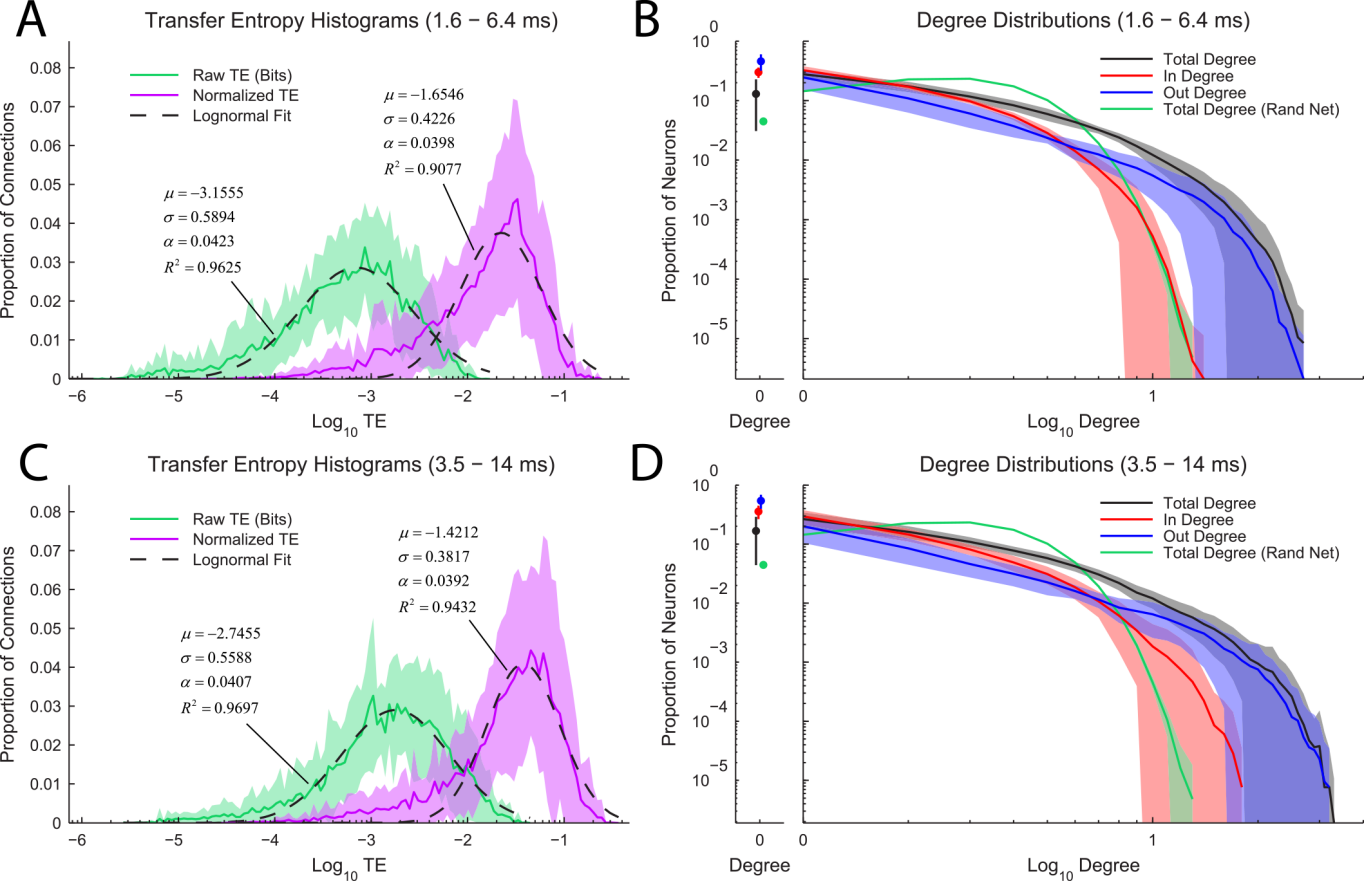
Transfer entropy and degree distributions. **(A and C)** Transfer entropy distributions for raw TE and normalized TE across the two time scales studied in this analysis (interactions with delays of 1.6–6.4 ms (A) and 3.5–14 ms (B)). Note that all distributions are roughly log-normal (see Eq 21, nonlinear regression performed in Matlab). Solid line is average of all recordings; shaded region represents ± one standard deviation across recordings. **(B and D)** In, out, and total degree distributions from the real data and total degree distributions from random networks with matching numbers of neurons, connections, and sampling statistics to the real data. Note that because the total degree distribution from the real data extends far beyond the distribution from the random networks, the real data are heavy-tailed. We did not assess whether the degree distributions were scale-free due to issues surrounding sub-sampling [93]. Also, note that the in-degree distribution had a shorter tail than the out-degree distribution, indicating that there were more high out-degree neurons than there were high in-degree neurons. Solid line is average of all recordings; shaded region represents ± one standard deviation across recordings.

Before analyzing the computations performed by neurons in the effective connectivity networks using multivariate TE, we first examined the distributions of bivariate TE values and the degree distributions of the networks (Fig 4).

Several previous studies found log-normal distributions of connection weights or distributions of weights that varied widely over several orders of magnitude [17–22]. Similar to these studies, we found a roughly log-normal distribution of significant TE weights (Fig 4A and 4C). We fit the distributions using the following probability mass function:

$$p\left(x\right)=\frac{\alpha }{\sigma \sqrt{2\pi }}e^{\frac{-{\left({\text{log}}_{10}\left(x\right)-\mu \right)}^{2}}{2\sigma^{2}}}$$ (21)

Note that we used a probability mass function because we binned the TE values into 100 logarithmically spaced bins to produce the distributions shown in Fig 4A and 4C. This binning necessitated the use of an additional normalization factor *α* in the fits. The TE distributions appeared more skewed when considering normalized TE values. This skew may be caused by the bound in the normalized TE at 1. Regardless of the precise distribution that best fits the TE distributions, both TE and normalized TE values varied broadly over several orders of magnitude.

We found the degree distribution from the real data to be markedly different from the degree distribution from random networks with identical numbers of neurons and connections (Fig 4B and 4D). Specifically, the degree distributions indicate that the real data contained many more high-degree neurons than would be expected in a random network. The random networks were created by randomly placing (uniform probability) binary connections in networks with numbers of neurons and numbers of connections set to match the real data. This result agrees well with previous studies that have found the degree distribution to be heavy-tailed in hippocampal networks [23] and cortical networks [15,24]. The subject of the precise nature of the degree distribution and whether it is scale-free has received a great deal of attention (e.g. [94]), but it has been shown that sub-sampled scale-free distributions are not scale-free [93]. Practically speaking, this implies that a perfect scale-free degree distribution would appear as a straight line in a log-log plot, but that a sub-sampled distribution would not simply be a noisy version of the ideal scale-free distribution. Rather, the sub-sampled distributions would appear bent. Our degree distributions appear to contain linear portions in log-log space, but they curve downwards at the end. We chose not to attempt to assess whether the deviations in these plots could be due to sub-sampling because of the wide range of assumptions that would be required. Rather, we felt it was appropriate to simply remark that the distributions from the real data are clearly heavy-tailed in that they contain more high-degree neurons than were found in random networks.

### Degree and Computation

**Fig 5:**
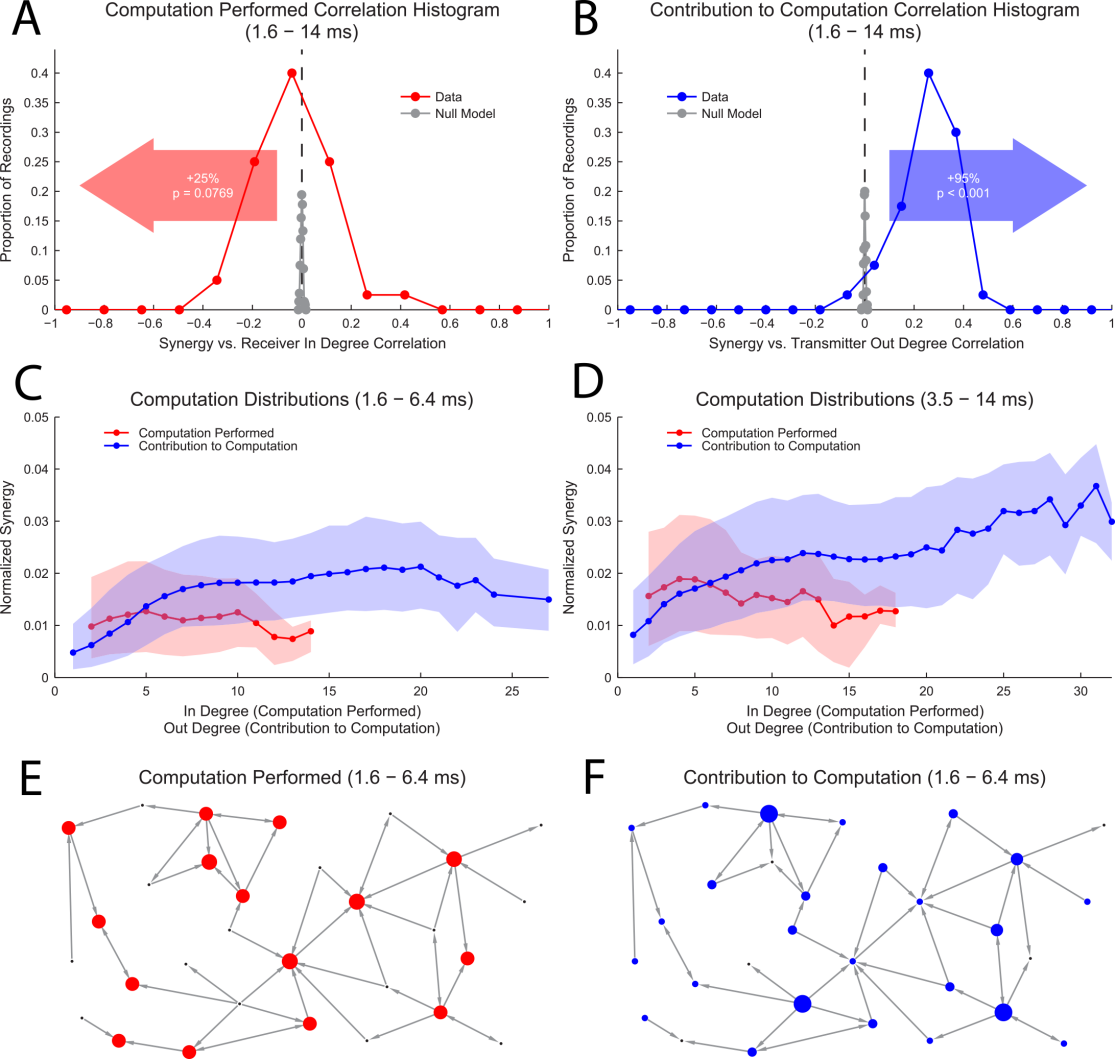
Degree-dependent computation. **(A and B)** Histograms across recordings of correlations between synergy (computation) and receiver in-degree (A) and between synergy (computation) and transmitter out-degree (B) (N_data_ = 40). Also shown is the skew towards positive or negative correlation values along with the likelihood to observe a skew of that magnitude or larger under the assumption positive and negative correlation values are equally likely (binomial cdf with p_pos_ = p_neg_ = 0.5). Nearly all correlations were likely to be significant given the proximity of the null model correlations (no correlation) to zero (null model consisted of randomized degree/computation pairings) (N_null_ = 400). Histogram bin size optimized using methods established in [95]. **(C and D)** Distributions of synergy values (computation) vs. degree averaged across all recordings. These plots show similar effects to (A and B). Solid line represents the median value; shaded region represents 1^st^ quartile to 3^rd^ quartile. Only degrees with 20 or more neuron groups are shown, so lower degrees, which had more neuron groupings, had a greater influence on correlation calculations in (A and B). Also, note that the in-degree distribution showed a shorter tail than the out-degree distribution (Fig 4B and 4D), so it was not possible to extend the computation performed plot to high in-degrees. **(E and F)** Explanatory computation performed (E) and contribution to computation (F) networks. In (E), notice that all neurons compute the same amount of information, but that in (F), neurons with high out-degrees contribute more information to computations. Dot size represents the median values from the matching degree in (C). This shows that computation was uncorrelated with the in-degree of the receiver neuron, but was correlated with the out-degree of the transmitter neuron.

Using recently introduced multivariate TE terms related to the partial information decomposition [3,4], we analyzed the amount of normalized information computed (multivariate synergy term) by individual neurons about the states of other neurons. For each neuron in the network that received two or more connections (defined by significant TE values), we calculated the computation (synergy) between each possible grouping of two input neurons and the receiver neuron (see Materials and Methods for details on the information calculations). After determining these computation values for all possible groupings of one receiver and two input neurons in the network, we calculated the correlation between the computation values and either (1) the in-degree of the receiver neuron or (2) the out-degree of one of the transmitter neurons (the other transmitter neuron being considered as a distinct data point) (Fig 5). We found two primary results. First, though significant positive and negative correlations between receiver in-degree and computation were found, the distribution of these correlation values for each recording was centered near zero with a slight negative skew (Fig 5A). Second, the majority of recordings exhibited positive correlations between transmitter out-degree and computation (Fig 5B). These trends were also observed in distributions of all neuron groups combined across all recordings for the two time scales of interest (Fig 5C and 5D). In general, these results imply that neurons tended to compute the same amount of bivariate information regardless of their in-degree (Fig 5E), but that neurons with high out-degrees tended to contribute more information used in computations by other neurons (Fig 5F).

**Fig 6:**
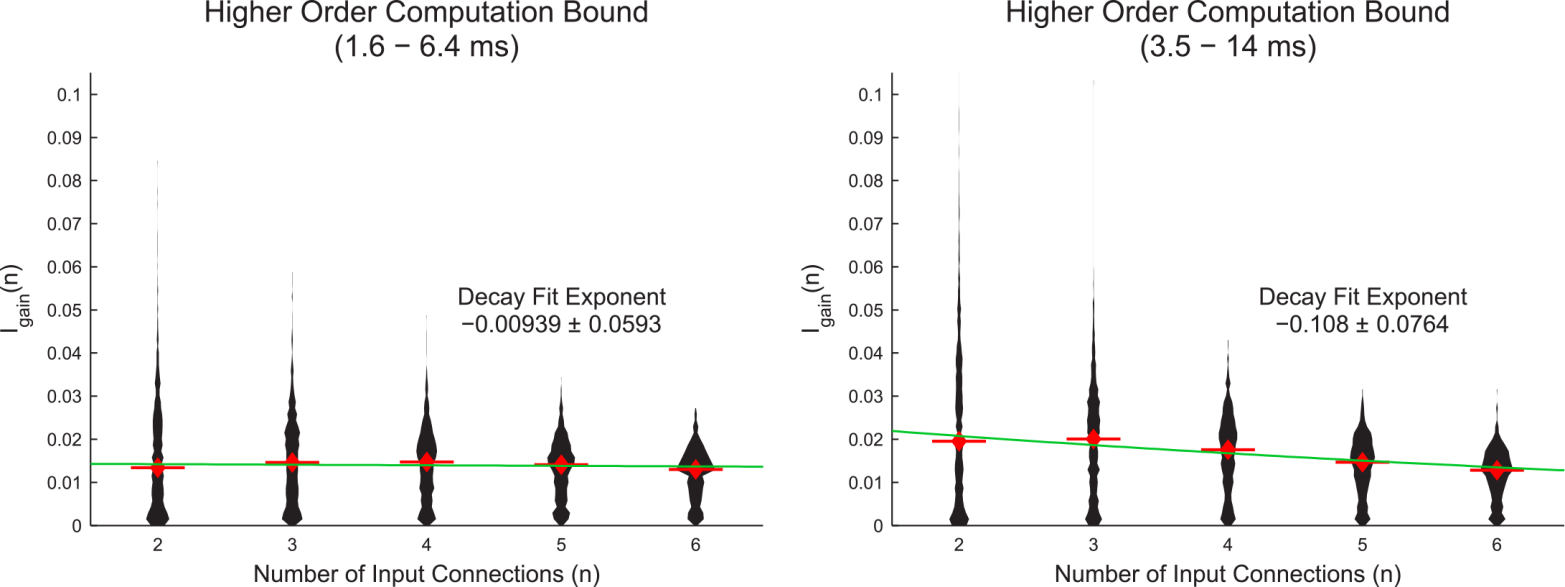
Higher-order computations did not dominate high in-degree neurons. Distributions of average information gains caused by adding more inputs for each neuron (I_gain_(n)) across all recordings (violin plots). Red bars and diamonds indicate medians. The n^th^ order synergy (computation) is less than or equal to the information gain. The median values were fit using exponential decay (green line). Negative exponents indicate decreasing information gains caused by adding more inputs. Both fits produced small negative exponent values, one of which had an error overlapping zero. (Fit: nonlinear regression performed in Matlab, Error: 95% confidence range in fit exponent value). These results indicate that higher-order computation tended to remain constant or decrease with added inputs.

Following the result that bivariate computation was independent of neuron in-degree, we chose to investigate higher-order computation terms. Unfortunately, higher-order computation is difficult to calculate [3], so we were limited to measuring a bound on the highest order computation using the information gain (see Materials and Methods). For the two time scales we examined in this analysis, we found that the information gain remained constant or decreased with added number of inputs (Fig 6). Because the information gain did not increase, we interpret these results to indicate that higher-order computation did not dominate high in-degree neurons.

### Feedforward Network Model

**Fig 7:**
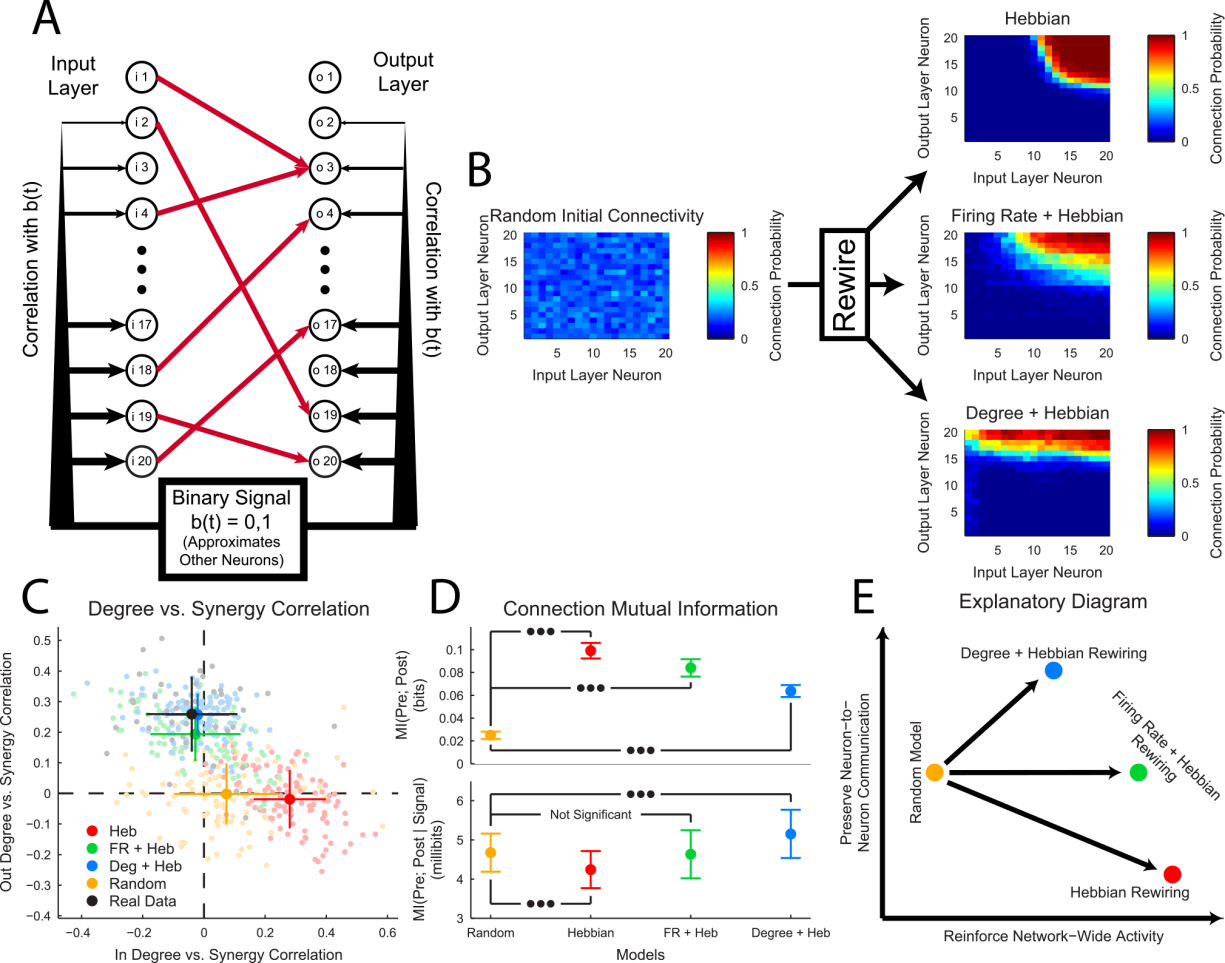
A degree-modified Hebbian rule qualitatively matched results from the original data and balanced reinforcement of network-wide activity with neuron-to-neuron communication. **(A)** Structure of the simple feedforward network model. Note that neurons with high indexes were more strongly correlated with the binary signal b(t). **(B)** Connectivity probability diagrams before and after network rewiring. Probabilities were averaged across 100 models. Note that the Hebbian rule pooled all the connections between neurons with strong correlations to the binary signal, while the degree-modified Hebbian rule preserved many connections from input layer neurons that were not highly correlated with the binary signal. **(C)** Degree vs. synergy correlation values for models and the real biological data. Note that the modified Hebbian rules qualitatively reproduced the correlation pattern seen in the real data. (Light dots represent individual models or recordings, dark dots represent mean value, and bars represent standard deviation) **(D)** Distributions of average mutual information between connected neurons (unconditioned (top) and conditioned on the binary signal (bottom)) across models. Note that the degree-modified Hebbian model showed higher mutual information after the effects of the common binary signal were removed. (mean value, bars represent standard deviation, Mann-Whitney rank-sum test (three dots: p < 0.001), False Discovery Rate Control [96–98]) **(E)** Though all rewiring methods increased the mutual information between connected pairs (which reinforces the common network activity defined by the binary signal), the degree-modified Hebbian rule also increased mutual information between connected neurons (neuron-to-neuron communication) independent of common network activity.

In order to better understand the degree vs. computation correlation results discussed above, we used a simple feedforward network model with various rewiring rules to produce degree vs. computation correlation results for comparisons to the results from the real data (Fig 7A). This model network was designed to capture a small segment of a larger network. The network consisted of two layers (input and output) of 20 neurons each. We included only 20 neurons in the model to reduce computation time. 80 binary connections linked input layer neurons to output layer neurons. Varying network-wide correlation was added to the network using a random binary signal that approximated the larger portion of the network. The presence of the binary signal further motivated the use of only 20 neurons in each layer of the network (see *Materials and Methods*for further details). Four distinct models were employed: a model with no rewiring and random connectivity, a model that used a Hebbian rewiring rule, a model that used a firing rate modified Hebbian rewiring rule, and a model that used a degree-modified Hebbian rewiring rule. The rewiring was controlled via a score calculated for all pairs of neurons (Eq 20, see *Materials and Methods*). The free parameters in the degree (*a*_1_ and *a*_2_) and firing rate (*a*_3_ and *a*_4_) modified Hebbian rules were found using a three stage manual lattice search of parameter space. This search was performed to find models that produced computation vs. degree correlation results that most closely matched the results seen in the real data. Therefore, the models represented different rewiring methods for fitting the computation vs. degree correlation results seen in the real data. The results of this search yielded the following score equations for the degree and firing rate modified Hebbian rules:

$$S_{Deg}\left(i,o\right)=A\left(i,o\right)+0.05Deg_{In}\left(o\right)-1.75Deg_{Out}\left(i\right)$$ (22)

$$S_{FR}\left(i,o\right)=A\left(i,o\right)+0.35FR\left(o\right)+3.1FR\left(i\right)$$ (23)

The three rewiring rules produced markedly different connectivity patterns (Fig 7B). As expected, the purely Hebbian rule pooled connections between the most strongly correlated neurons (correlation created via the binary network-wide signal). The firing rate modified rule spread connections more broadly to input layer neurons less well correlated with the binary network-wide signal. Finally, the degree-modified rule, primarily by virtue of the strong negative weight associated with the degree of the input layer neurons (Eq 22), spread connectivity almost uniformly across the input layer.

Both the degree-modified and firing rate modified Hebbian rules qualitatively matched the degree vs. computation correlation results seen in the real data (Fig 7C). The degree-modified rule was closer than the firing rate modified rule, but given the wide differences between the model and the real cortex, the significance of the correlation matching results cannot be assessed. That said, the purely Hebbian rule and the random network produced correlation results that were qualitatively very different from the real data.

Next, we compared mutual information (Eq 1) between connected neurons in the four models (Fig 7D). Note that we did not examine the locations of the connections (see Fig 7B) or whether connections were moved in assessing information transmission along connections. Rather, we examined the amount of information transmitted through network connections regardless of the locations of the connections. Unsurprisingly, we found the largest mutual information in the purely Hebbian rule. This was expected because the Hebbian rule pooled connections among already strongly correlated neurons. However, when we conditioned the mutual information on the binary network-wide correlation signal, we found that the degree-modified Hebbian rule produced the highest mutual information. Because mutual information is a measure of communication [56], we can view the conditioned mutual information as neuron-to-neuron communication in the presence of network-wide correlations and we can view the unconditioned mutual information as primarily carrying information about network-wide correlations. Therefore, the degree-modified rule–which produced degree vs. computation results that most closely matched the real data–both increased neuron-to-neuron communication and reinforced network-wide correlations, while the other rewiring rules maintained or decreased neuron-to-neuron communication (Fig 7E).

Our model was only intended to serve as an illustration of the computation results found in the biological tissue and therefore results from the model cannot be expected to directly relate to cortical networks. Though, to insure that our results were not heavily dependent on network size, we ran the network model with 40 neurons (double the original size) to see how the number of neurons affected the overall model results (S1 Fig). This new model produced the same firing rate modified Hebbian rewiring rule (Eq 23) and a similar degree-modified Hebbian rewiring rule given by:

$$S_{Deg,40}\left(i,o\right)=A\left(i,o\right)+0.16Deg_{In}\left(o\right)-0.3Deg_{Out}\left(i\right)$$ (24)

The new model produced connectivity diagrams (S1B Fig) qualitatively identical to those seen in the smaller model (Fig 7B). As with the smaller model, the larger model produced computation correlation results similar to the results seem in the data for the degree-modified and firing-rate modified Hebbian rewiring rules, but not the pure Hebbian rewiring rule (S1C Fig). Results for the connection mutual information in the larger model were very similar to the results seen in the smaller model, though the Hebbian and firing rate modified Hebbian rewiring rules produced larger conditioned mutual information than was seen in the small model (S1D Fig). As in the smaller model, the degree-modified Hebbian rewiring rule reinforced network-wide activity and increased neuron-to-neuron communication (S1E Fig).

## Discussion

### Main Finding

The main finding of this work is that the amount of information computed by a neuron about the states of other neurons depends significantly on its topological location in the surrounding functional network. More specifically, the neurons that compute the most information tend to receive inputs from high-degree neurons. The in-degree of a neuron, however, has no relationship to the amount of information it computes.

### Connectivity Weight and Degree Distributions

Previous studies have found a log-normal (or at least widely varied) distribution of synaptic weights in networks of cortical neurons [17–21]. The presence of a wide range of connection strengths dictated by a log-normal distribution can significantly impact several features of a network, including signal propagation in the presence of noise and synaptic plasticity [20]. We found a roughly log-normal distribution of transfer entropy values. It is interesting that both structural connectivity as assessed in these previous studies and the effective connectivity measured herein produced similar distributions of connection weights. Though such experiments are currently difficult to perform, future studies could be conducted to further investigate the relationships between these two types of connectivity (see [42] as an example of such an analysis conducted in a model).

In our analysis of the degree distributions, we found them to be heavy-tailed, indicating that some neurons had more connections than would be expected if the network were randomly connected. This result corresponds well with different analyses performed using the same data [15,24] and previous studies conducted in hippocampus [23]. The nature of the degree distribution has been widely discussed in the literature, with possible implications including network formation mechanisms and rhythm formation [94,99]. Due to issues surrounding sub-sampling, we were unable to determine if the degree distributions were actually scale-free [93]. Still, the presence of high-degree neurons leads to the question of what role degree plays in the network, which we primarily addressed by examining computation (see below).

### Degree/Computation Relationship

We sought to measure the relationship between the neuron degree and computation performed or contributed. Recall, we defined computation using the synergistic information calculated using the PID because this portion represents the information gained by simultaneous knowledge of all input variables. We found that computation performed, as defined here, was relatively independent of in-degree, while contribution to computation was correlated with out-degree. This indicates that neurons that received more connections did not compute more information than neurons that received a few connections. Conversely, we found that neurons that sent out many connections contributed more information to computations performed by other neurons. In other words, we found that neurons that received connections from high out-degree neurons tended to compute more information from those high out-degree neurons than did neurons that received connections from low out-degree neurons.

There are several interesting consequences of these results. First, because we primarily measured bivariate computation (two neurons sending connections to a third), it is possible that neurons with high in-degrees were performing higher-order computations. We addressed this issue by calculating a limit on the highest order synergy term (highest order computation). This analysis showed that the limit of the higher-order computation was constant or decreasing as the number of inputs is increased. We interpret this result to indicate that higher-order computation did not dominate high in-degree neurons. However, our analysis only placed a limit on the highest order computation and, due to the large number of synergy terms for many input variables, it is possible that for some neurons certain terms were maximized for large numbers of input connections. Therefore, we feel these higher-order computations must be investigated further. These higher-order computation effects may be especially relevant for studies of conductance states in high in-degree neurons [18,100].

Second, the correlation between neuron out-degree and contribution to computation implies that high out-degree neurons have a special role in the network. It appears that these neurons were broadcasting information the rest of the network was using in computations, so perhaps they were sources of especially relevant or important information. Furthermore, it is possible that these neurons were physiologically different from other neurons (e.g. excitatory, inhibitory, located in a certain cortical layer, etc.) and/or that there was some type of interplay between the information the neuron provides and how it formed connections with other neurons. Unfortunately, we were unable to investigate either of these possibilities in this experimental system, though we hope to do so in the future.

Third, the lack of correlation between neuron in-degree and computation performed implies that neurons compute a similar amount of information regardless of their in-degree. This even spreading of the computational burden may be the most robust or efficient. Alternatively, this result may be due to spike rate limitations in the neurons themselves or to the presence of higher-order computations (see above). Additionally analyses could be undertaken in the future to assess the role spike rate limits may play in neuron computation. Also, it may be possible that high and low in-degree neurons, while computing the same amount of information, are performing different types of computations. Perhaps some types of computations are best performed by low in-degree neurons, while other types are best performed by high in-degree neurons. Additional studies could be conducted to characterize the types of computations performed by the neurons. Finally, we did not relate the information computed by a neuron to its out-degree. So, while in-degree did not affect the amount of information computed by a neuron, neurons that computed a large amount of information may have sent out more connections than neurons that computed a small amount of information. In the future, we hope to examine this possibility with further analyses.

Fourth, in this analysis we related computation to neuron in and out-degree, but it would be interesting to relate computation to other network topology metrics [101], such as modularity [102], assortativity [103], and the clustering coefficient [104]. Perhaps computation primarily occurs in neurons that receive connections from distinct modules. Also, the relationships we found between computation and degree may have a special importance for network assortativity given the emphasis placed on the degrees of connected neurons in the assortativity calculation.

### Feedforward Network Model

Using a toy feedforward network model, we found that it was possible to tune a degree-modified Hebbian rewiring rule to produce computation vs. degree correlation results that qualitatively matched the results seen in the real data. We then compared this tuned degree-modified Hebbian rewiring rule to other possible rules. We found that a purely Hebbian rewiring rule and a random network produced computation vs. degree correlation results that were markedly different from the results found in the real data. Furthermore, we found that the degree-modified Hebbian rule maximized neuron-to-neuron mutual information in the presence of network correlations, while still increasing reinforcement of network-wide correlations. Finally, the specific parameter values that produced the degree-modified Hebbian rule included a negative weight for the out-degree of the input layer neurons. This negative weight had the effect of spreading connections broadly from input layer neurons, which may be relevant for recent studies of bottlenecks in network activity [105].

Obviously, the simplicity of this model prevents us from drawing direct conclusions about networks of neurons in the cortex from our results. We wish to emphasize that in a more realistic model or in the actual cortex, contradictory results could be found. For instance, our model did not contain inhibitory connections, which would probably greatly affect firing rate modified Hebbian rules. In spite of that, the results from our model do motivate several intriguing hypotheses that should be investigated further. First, our result that a degree-modified Hebbian rewiring rule best matched correlation results opens the door to exploring degree-modified Hebbian wiring rules in more accurate models and organic systems. We are unaware of any other analysis that has directly incorporated neuron degree in a Hebbian rewiring rule. Though a similar concept (preferential attachment [92]) has been explored in the literature. Because it seems unlikely that neurons have direct access to information about their degree or the degrees of other neurons, experiments should be performed to see if some other physiological or chemical factors are capable of communicating this information. That said, one could imagine possible explanations for why neurons with many connections would tend to gain more connections (e.g. to spread relevant or important information) or why neurons with many connections would tend not to gain more connections (e.g. fault tolerance, resource allocation concerns, etc.). Similar hypotheses could be developed for firing rate modified Hebbian rewiring rules. Second, our result that the degree-modified Hebbian rewiring rule also maximized neuron-to-neuron communication in the presence of network-wide correlations while simultaneously increasing the reinforcement of network-wide correlations points to possible relationships between signal propagation in correlated networks, computation, and network topology. Furthermore, it is noteworthy that all three rewiring rules tended to concentrate the end point of connections in neurons that possessed strong network-wide correlation, but the degree-modified and firing rate modified Hebbian rewiring rules spread the start point of connections to neurons that were not strongly correlated with network-wide correlations. Given the wide interest in the topic of signal propagation in correlated and noisy networks [57–63], this result should be further investigated. Perhaps network topology is determined in such a way that computation and information transmission are routed through certain neurons, while other neurons maintain network-wide correlations. We hope to further investigate these issues in more accurate models and *in vivo* systems in the future.

### Unutilized PID Multivariate TE Terms

For the sake of simplicity, we chose to only focus on the synergy term (computation) for this study. However, the other PID terms could provide useful information about the cortex. For instance, though unexplored here, the PID multivariate TE redundancy term may prove useful for measuring interactions from correlated inputs. It may be the case that high out-degree neurons tend to broadcast more redundant information than low out-degree neurons, implying that network-wide correlations may be managed by high out-degree neurons. Also, the redundancy term could be used to group neurons into functionally similar groups. It would be interesting to relate these functionally similar groups to topological properties, such as degree and modularity [101,102,106], and, if possible, to neuron properties like cell type or physical location in the tissue. Finally, it would be interesting to compare PID multivariate TE unique information terms to redundancy and synergy, especially for higher-order interactions if possible. Doing so would illuminate the contributions from individual neurons in the network. It is possible that the unique and synergistic terms decrease with additional inputs, while the redundancy terms increase, thereby possibly reducing the importance of individual neurons in the network. In the future, we hope to further investigate these terms in the cortex and other systems, and we would like to emphasize that these other terms may be crucial to additional analyses of the topics discussed above.

### Limitations and Strengths of the Analysis

Perhaps the most noticeable potential limitation of this analysis is the fact that it was performed using organotypic cultures [67,107]. Although organotypic cultures have been widely used in research [108,109], these cultures have been shown to possess several differences in comparison to the *in vivo* system using both mice and rats. Such differences *in vitro* include additional synaptic connectivity [110,111], decreased ease of LTP induction [112], changes in protein expression [113], increased excitability [111,114], and changes in cellular organization in mice [115].

Despite these issues, the overall structure and electrical activity of cortico-hippocampal organotypic cultures have been shown to essentially match the *in vivo* system [110,112,116]. Furthermore, it has been shown that interneurons in organotypic cultures are physiologically and morphologically identical to interneurons *in vivo* [117], cortical layer structure and cell migration are preserved in postnatal organotypic cultures (as were used in this analysis) in rats [118], and that intracortical connection structure is preserved in organotypic cultures when sub-cortical regions are preserved in culturing (as was done in this analysis) [119–121].

Based on these previous studies, we concluded that organotypic cultures represent a useful model system for intact *in vivo* neural systems. Therefore, we believe our results are relevant for the field given the strength of the preparation used, the power of the analysis, and the novelty of the results themselves. Furthermore, at this time, it would not have been technologically possible to achieve the same level of spatial and temporal recording resolution and the same number of recorded neurons *in vivo*. While some *in vivo* recording methods are capable of recording from hundreds of neurons, these methods demand trade-offs in terms of temporal or spatial resolution. For instance, *in vivo* calcium imaging allows for the simultaneous recording of up to approximately 1000 neurons, but the temporal resolution for these recordings is significantly less (tens of ms) than we achieved in our recordings (50 μs) [122–124]. Recording methods with lower temporal resolution would have been unable to capture the interactions that we observed. Furthermore, *in vivo* electrophysiological recording methods that employ planar arrays or shank electrodes are capable of recording hundreds of neurons with high temporal resolution, but these recording methods possess limited spatial resolution in comparison to our array (inter-electrode spacing of 60 μm) due to larger inter-electrode spacing in arrays (e.g. 400 μm in Utah arrays (Blackrock Microsystems)) and larger spacing between shanks (e.g. 250 μm in [125]). Therefore, our use of organotypic cultures and a high density, high temporal resolution multi-electrode array permitted a dramatic improvement in the quality of the data, which improved the strength of the analysis.

Our recording method possessed several distinct features that were advantageous especially during the developmental stages of this method. Still, other recording methods could be used with this analysis method to investigate other phenomena. For instance, the use of *in vivo* calcium imagining, while lacking the temporal resolution to capture short time scale connections, would more easily facilitate the gathering of additional information about the neurons involved in the networks (e.g. cell type, cell layer, physical location in the tissue, etc.). In this analysis, we related computation to the topological locations of the neurons in the functional networks, but we were unable to relate neuron computation to the physical locations of the neurons in the tissue. Also, *in vivo* calcium imaging would more easily allow for direct cell stimulation or inhibition via optogenetic techniques [126]. Furthermore, *in vivo* calcium imaging reduces the burden associated with spike sorting because the neurons are visually identified. Spike sorting in any analysis is a significant issue because it typically prevents detection of neurons with very few spikes. In our analysis, for instance, we only recorded ~300 neurons on average from each culture despite the fact that our array covered approximately 2 mm^2^ of tissue. (For comparison, we estimate roughly 10,000 neurons were covered by the array.) While the issue of large populations of silent or nearly silent neurons is itself an active area of research [127], utilizing a method like *in vivo* calcium imaging could reduce problems with low spike count neurons. Finally, *in vivo* studies could investigate the relationship between computation and phenomena that can only be studied *in vivo*, such as behavior and sensory coding (e.g. [22]). We feel these types of analyses could produce novel insights into computation at the cellular level in the brain and we plan to pursue them in the future.

In addition to new questions that could be addressed with different types of experimental systems and recording methodologies, improvements can also be made to the information analysis method itself. First, of particular relevance to the analysis method is how it addresses noise. In this analysis, we used randomized data that preserved noise to some extent in the process of generating null information values. We then compared the distribution of null information values to the real values to establish significant TE [44]. We did not characterize how different types of noise or levels of noise affect this method, nor does the method explicitly take noise into account. In the future, we hope to improve the information analysis method to explicitly incorporate noise. Second, though we were able to calculate a limit on higher-order synergy terms (Fig 6), the PID analysis method is difficult to scale up to more than three neurons. These higher-order interactions are most likely very relevant in neural networks. Though, complex interactions between many variables are notoriously difficult to analyze with any tool, so this limitation of the PID analysis is not unique and future work must be done in general to address the topic of higher-order interactions. Furthermore, two or three neuron interactions (as were studied herein) can be successfully analyzed using the PID. Another limitation of this analysis is that we examined two combinations of bin size and delay, but other bin sizes and delays could be used, possible complicating the analysis. In the future, we hope to develop methods to address these issues and they represent important concerns for other analyses.

## Supporting Information

S1 Fig Large feedforward model network results. All subfigures in this panel correspond to the subfigures in Fig 7. This model contained 40 neurons per layer (double the original network size), but otherwise matched the smaller model in terms of set parameters and equations. Note that the larger model produced connectivity diagrams and computation correlation results **(B and C)** that were very similar to the smaller model (Fig 7B and 7C). Also, note that the larger model produces similar mutual information results **(D)**, with the exception of the Hebbian and firing rate modified Hebbian rewiring rules for the conditional mutual information (Fig 7D). As with the smaller model (Fig 7E), the degree-modified Hebbian rewiring rule reinforced common network activity and it increased neuron-to-neuron communication independent of the common network activity **(E)**.
(EPS)
